# Disposable Food Packaging and Serving Materials—Trends and Biodegradability

**DOI:** 10.3390/polym13203606

**Published:** 2021-10-19

**Authors:** Katarzyna Dybka-Stępień, Hubert Antolak, Magdalena Kmiotek, Dominik Piechota, Anna Koziróg

**Affiliations:** 1 Faculty of Biotechnology and Food Sciences, Institute of Fermentation Technology and Microbiology, Lodz University of Technology, Wolczanska 171/173, 90-530 Lodz, Poland; hubert.antolak@p.lodz.pl (H.A.); 220988@edu.p.lodz.pl (D.P.); anna.kozirog@p.lodz.pl (A.K.); 2 Centre of Papermaking and Printing, Lodz University of Technology, Wolczanska 223, 90-005 Lodz, Poland; magdalena.kmiotek@p.lodz.pl

**Keywords:** food packaging, disposable tableware, single-use cutlery, eco-friendly utensils, edibles, bioplastics, biopolymers, biodegradability

## Abstract

Food is an integral part of everyone’s life. Disposable food serving utensils and tableware are a very convenient solution, especially when the possibility of the use of traditional dishes and cutlery is limited (e.g., takeaway meals). As a result, a whole range of products is available on the market: plates, trays, spoons, forks, knives, cups, straws, and more. Both the form of the product (adapted to the distribution and sales system) as well as its ecological aspect (biodegradability and life cycle) should be of interest to producers and consumers, especially considering the clearly growing trend of “eco-awareness”. This is particularly important in the case of single-use products. The aim of the study was to present the current trends regarding disposable utensils intended for contact with food in the context of their biodegradability. This paper has summarized not only conventional polymers but also their modern alternatives gaining the attention of manufacturers and consumers of single-use products (SUPs).

## 1. Introduction

The European Union’s Directive (EU) 2019/904 of the European Parliament and of the Council [1] on the reduction of the impact of certain plastic products on the environment describes single-use plastic products (SUPs) as materials that are used once, or for a short period of time, before being thrown away. On the other hand, this can be complemented by the definition of SUPs as products that have been made entirely or partially of plastic but are not designed to be used multiple times by, e.g., being returned to the manufacturer for re-entering the market. The group of these products includes, among others, cutlery, plates, straws and stirrers, food and beverage containers, cups for beverages, packets and wrappers, and plastic bags. The main purpose of the implementation of the directive is to prevent and reduce the impact of plastics on the natural environment, and thus human health.

The beginning of industrial production of plastic is considered to be the 1950s. Since then, the global production of plastics increased from 1.5 to 259 (2018), 368 (2019), and 367 (2020) million metric tons [2]. The main producers of plastic in the world are Europe (17%), North American Free Trade Agreement (NAFTA) countries (18%), and Asia (51%). It is worth noting that China alone is responsible for 30% of the world’s plastic production. China’s plastic exports have grown considerably from USD 14.4 billion in 2009 to USD 48.3 billion by 2019. One of the industries that contributes to the growing production of plastics is the food industry and related areas, such as restaurants and street food vendors. Nearly 40% of plastic in the European Union is used for packaging goods, including food products. Globally, in 2016, 485 billion polyethylene terephthalate (PET) bottles were produced, and it is predicted that in 2021 approximately 583 billion of these plastic bottles will be produced. In the United States of America, approximately 500 million single-use plastic straws are used each day, while in Europe it is about 25.3 billion in a year. Continuing, the plastic cutlery market worldwide in 2017 was valued at approximately USD 2.6 billion. The features that make plastics highly desirable are a curse for the environment. It is believed that the world is literally flooded by plastic, and only 9% of synthetic polymers produced are recycled.

In a relatively short period of time (1950–2020), the global production of plastics as well as their irresponsible use and the lack of sustainable waste management has caused their occurrence in hardly accessible places: from high parts of mountains to the bottom of seas and oceans; the name of a newly described amphipod found at a depth of 6900 m—*Eurythenes plasticus*—comes from the plastic found in its gut [3]. The presence of these polymers in extreme environments is the result of their defragmentation, which is due to the fact that the vast majority of polymers currently used do not degrade, but instead slowly fragment into smaller and smaller pieces and particles. The smallest know fragments of plastics are called microplastics, but most recent discussions consider the use of the term “nanoplastics” [4]. Both in the form of larger pieces as well as microparticles, plastic is commonly found in the stomachs of numerous wild animals (birds, turtles, dolphins) as well as in livestock, where it is transferred to the human body through the digestive system. In addition, substances that are used to improve the parameters of plastics (plasticizers) may enter organisms, where they bioaccumulate.

The global problem of the over-production of plastics (including those used for food consumption), environmental pollution, and the growing number of studies and publications on the negative impact on consumer health along with growing awareness of the consumers on these negative aspects has brought the world to global changes in terms of single-use materials, their production, use, and waste management. For example, strategies of the limitation of SUP’s use are focusing on (1) prohibition on placing plastic cutlery and plates, beverage stirrers, and beverage containers on the market; (2) a reduction in the consumption of single-use plastic products (cups for beverages and specific food containers); (3) separate collection through the implementation of deposit-refund schemes; (4) reduction of the consumption through awareness-raising measures; (5) labeling of products to inform about the content of polymers and the damage they can cause in the natural environment; (6) introduction of extended producer responsibility systems in the field of waste management; (7) introduction of natural, ecological equivalents of previously used cutlery, knives, forks, plates, and cups made of natural raw materials. The last of these issues is particularly interesting from a scientific point of view. The results obtained in digital literature databases such as Web of Science show the growing interest in the application of natural polymers in the production of single use tableware and cutlery.

**Figure 1:**
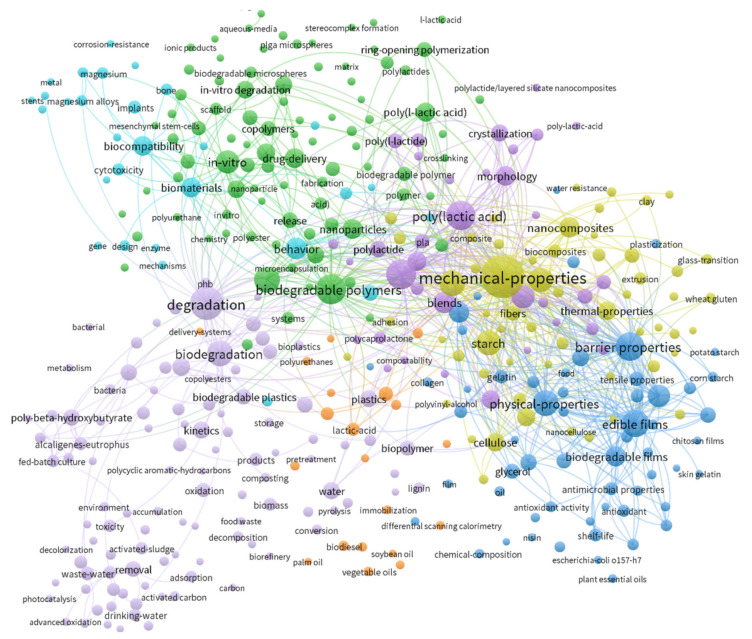
Co-occurrence analysis of the data from Web of Science obtained for 13,292 records. Bubble size presents the number of papers in the database. Bubble proximity presents frequency of co-occurrence of phrases in the same papers.

Web of Science (WoS) was accessed in September 2021 in order to identify and analyze relevant publications, books, and other documents. The following combinations of key words were applicated: “single-use” AND (“tableware” OR “cutlery” OR “utensils” OR “cups” OR “plates” OR “bowls” OR “knives” OR “forks” OR “spoons” OR “chopsticks” OR “stirrers” OR “meal-boxes” OR “packets” OR “wrappers”) (123 records from WoS). For “edible” AND (“tableware” OR “cutlery” OR “utensils” OR “cups” OR “plates” OR “bowls” OR “knives” OR “forks” OR “spoons” OR “chopsticks” OR “stirrers” OR “meal-boxes” OR “packets” OR “wrappers”) 182 records from WoS were given. On the other hand, the combination “edible” AND (“coatings” OR “films” OR “packaging” OR “materials”) gave 10,928 records, while for “biodegradable” AND (“plastics” OR “packaging” OR “tableware” OR “cutlery” OR “utensils” OR “products”) 13,292 records were found (Figure 1). What is more, for “single-use” AND (“polyethylene” OR “polyvinyl chloride” OR “polyethylene terephthalate” OR “polystyrene” OR “polyethylene furanoate” OR “polybutylene succinate” OR “biocomposites” OR “PLA” OR “polylactic acid” OR “PET” OR “PS” OR “PEF”) 335 records, while for “packaging materials” AND (“polyethylene” OR “polyvinyl chloride” OR “polyethylene terephthalate” OR “polystyrene” OR “polyethylene furanoate” OR “polybutylene succinate” OR “biocomposites” OR “PLA” OR “polylactic acid” OR “PET” OR “PS” OR “PEF”), 1746 records were assumed. The combination of (“cutlery” OR “tableware” OR “utensils”) and (“polyethylene” OR “polyvinyl chloride” OR “polyethylene terephthalate” OR “polystyrene” OR “polyethylene furanoate” OR “polybutylene succinate” OR “biocomposites” OR “PLA” OR “polylactic acid” OR “PET” OR “PS” OR “PEF”) gave 89 records.

## 2. Disposable Tableware and Cutlery—Categories and Classification

**Figure 2:**
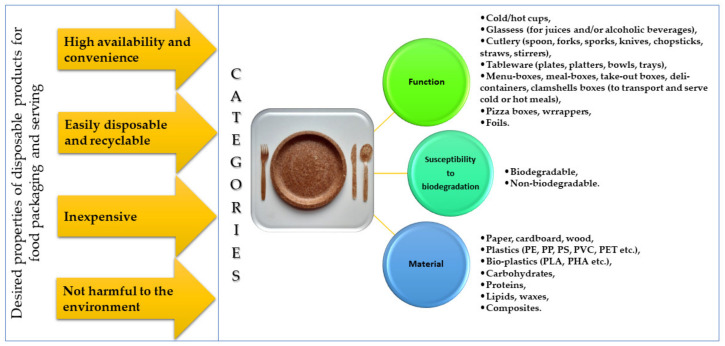
Classification of single-use tableware and cutlery.

Urbanization requires food to be easily available, stored, and transported. Recently, eating out of home has increased significantly. According to the Eurostat [5], in 2018, households in the European Union (EU) spent over €600 billion on “catering services”: restaurants, cafés, canteens, catered events, and the like. The food service industry faces a huge challenge in the 21st century, because a large portion of restaurant orders are take-away or delivered. The COVID-19 pandemic contributed to the enormous increase in the amount of takeaway orders [6]. It should be highlighted that the mass consumption of disposable plastic tableware increases drastically during the summer months, especially during the season of picnics, barbecues, and music as well as food-truck festivals. Another important sector is the civil aviation industry, which requires huge amounts of food packaging materials. Simultaneously, the consumption of ready-to-eat convenience food as well as dietary catering have gained considerable interest in recent years, especially in the context of independent lifestyles and an increasing number of people living alone. Diverse categories to classify single-use food packaging are summarized at the Figure 2 along with the properties that make them attractive to consumers. By product, the single-use tableware and cutlery market can be categorized into cups, glasses, spoons, forks, sporks, chopsticks, straws, etc. Single-use packaging materials can also be segregated into biodegradable and non-biodegradable with separate subcategories (e.g., compostable, edible) resulting from the type of raw material. Additionally, segregation by commercial and household applicability can be mentioned, but in many cases, the main difference is in the number of items sold in the collective packaging.

**Figure 3:**
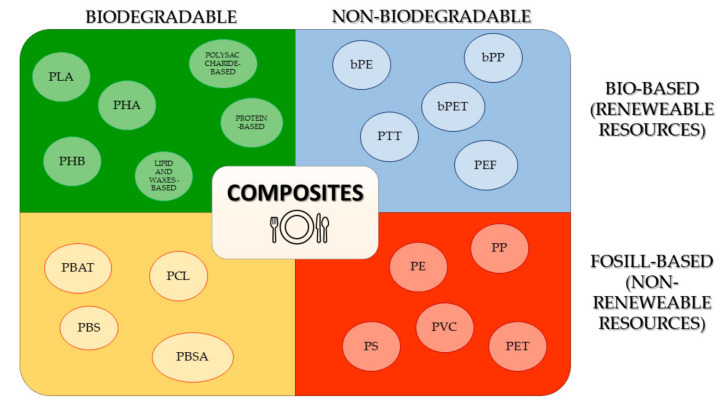
Diagram positioning (bio)plastics used in production of single-use tableware and cutlery (own study based on Mendes and Pedersen [9]). Abbreviations: PLA (polylactic acid), PHA (polyhydroxyalkanoate), PHB (polyhydroxybutyrate), PE/bPE (polyethylene/renewable resource-based polyethylene), PTT (polytrimethylene terephthalate), PET/bPET (polyethylene terephthalate/renewable resource-based terephthalate), PP/bPP (polypropylene/renewable resource-based), PEF (polyethylene furanoate), PBAT (polybutylene adipate terephthalate), PCL (polycaprolactone), PBS (polybutylene succinate), PBSA (polybutylene succinate adipate), PVC (polyvinyl chloride), PS (polystyrene).

Although the polymers most frequently used for the production of disposable and single-used plastic are recyclable, only a small fraction of them, not exceeding 9%, is subjected to recycling [7]. Emerging climate change and the theory of circular economy has forced the application of the recycling system in the field of wastes from food packaging; however, in many countries, it is still not formed or is not yet perfected. On the consumer side, there is no effective recycling mechanism, and most of the takeaway waste is directly thrown into the trash bin and disposed of in landfills without any recycling. Along with the higher eco-awareness regarding the microplastic or nanoplastic problem (supported with the legacy initiatives), producers have offered a wider portfolio of eco-friendly and biodegradable tableware and utensils and consumers have started to choose them as more sustainable products. Single-use tableware and cutlery can be produced with a wide variety of materials. These materials can be of virgin or recycled origin. They can be obtained by simply shaping it to desired form (for example, wood-based utensils or flatware) as well as after a minor or highly complex processing, including, for example, preparation of molded composites of natural fibers (or other fillers) and (bio)plastics or totally (bio)-plastic items. Figure 3 presents a large family of the (bio)plastics materials used for this purpose [8].

The term “bioplastics” is commonly used in two contexts, which can be misleading. According to the European Bioplastic Organization [10], a bioplastic is material derived (at least partly) from renewable resources (biomass, microorganisms, etc.). The second connotation of this term refers to its biodegradability, and in this context, “bioplastics” are considered as a synonym of “biodegradable materials”.

In general, five main categories of (bio)plastics-derived material used for the production of disposable utensils and cutlery can be defined as follows: (1) biodegradable and bio-based, (2) non-biodegradable but bio-based, (3) possessed from non-renewable sources but biodegradable, (4) fossil-based and non-biodegradable, (5) composites of blended miscellaneous (bio)plastics, which can be also mixed with fillers of different origin (Figure 3). Their origin, and detailed characteristics will be described in the following sections of the paper.

According to the European Bioplastics [11], bioplastics represent about one percent (4.2 million tons) of the more than 368 million tons of plastic produced annually but their ratio constantly increases. Simultaneously, packaging was the largest area of application for bioplastics with 47% of the total b ioplastics market reported in 2020. The choice to use bioplastic disposable materials contributes to ensuring more sustainable products for food packaging and serving. In recent years, for the United Nations Environment Programme, reports referring to Life Cycle Assessment (LCA) for several categories of single-use plastic-based products were prepared and published. Two of them refer to the detailed LCA description of single-use plastics: takeaway food packaging and its alternatives [8] and single-use plastic tableware and its alternatives [12]. These reports summarize the findings of the analysis, including some of the environmental benefits and drawbacks of the production, maintenance, and utilization of these products. When designing a new product for commercialization, all of the aspects of its LCA including analysis of its features referring to degradation rates in various conditions, changes in mechanical and optical properties during storage, microbiological safety and the possibility of releasing harmful compounds to the packaged and served food should be taken into account.

The next section of the article, “Conventional materials used in the production of dishes and cutlery”, describes a wide spectrum of classical polymers (in the meaning of fossil-based, and mostly non-biodegradable) and bio-based, biodegradable polymers (including natural and synthetic polymers) and their composites used in food packaging and service with single-use products; in Section 4 and Section 5, new and interesting solutions invented for the production of single-use tableware and cutlery made of wastes from the agro-food industry and edible cutlery and utensils have been discussed, respectively.

## 3. Conventional Materials Used in the Production of Dishes and Cutlery

### 3.1. Classical Polymers

Of the total plastic production, 42% is used for packaging, 19% for construction, and 17% for the textile industry [13]. The main primary polymers produced worldwide are polypropylene (PP), low-density polyethylene (LDPE), polyester, acrylic or polyamide fibers, high-density polyethylene (HDPE), polyvinyl chloride (PVC), polyethylene terephthalate (PET), polyurethanes (PUT), polystyrene (PS), and additives [14]. In packaging dedicated to the food industry, mainly LDPE, PP, HDPE, PET, PS, PVC, and EPS (expanded polystyrene) are applied.

In 2019, in the European Union, packaging constituted 39.6% of plastics production with the highest consumption of PP, LDPE, HDPE, PVC, PET, and PS (together with EPS) in the given order [15]. The demand on the above-mentioned polymers accounted for 19.3%, 17.5%, 12.2%, 10%, 7.7%, and 6.4%, respectively, of all primary plastics manufactured [16].

#### 3.1.1. Polypropylene

Polypropylene (PP), with ca. 10 M tons, is at the top of the plastics converter demand list [16]. However, its production volume makes it the world’s second-largest plastic resin. In 2018, its production reached 56 million metric tons, and almost a doubling of this value is predicted to the year 2026 [17]. Increasing production of PP is caused by its good properties allowing for versatile use with simultaneous ease of processing with numerous methods dedicated for thermoplastics.

Polypropylene is produced in anion, cation, or radical polymerization from gaseous propylene with the application of stereospecific Ziegler–Natta catalyzers. In the end, stereoregular polymer, with chiral centers on carbon atoms bonded to methyl groups, is formed. Stereoregular PP can be atactic (irregular), syndiotactic (alternating), or isotactic (regular) from the point of view of the methyl groups position in relation to the main chain.

The best properties of PP, especially in terms of excellent tensile strength and stiffness, are assigned to isotactic form. Commercial propylene polymers are mainly isotactic and contain no more than 5% of the atactic form, which is amorphous and tacky, and is used mostly as a hot-melt adhesive. Syndiotactic PP contains less content of crystalline phase and has limited use, mainly as an elastomer. Only isotactic polypropylene, due to its semi-crystalline nature and other properties as a consequence of its high crystalline phase content, is applied as a commercial plastic for food packaging, pipes, fittings, carpets, and large molded parts for automotive and consumer products. PP is also used for the production of fibers for durable lines, fishing nets, and filter fabrics used in the chemical, food, and ceramic industries [18,19]. Polypropylene is the preferred type of polymer for rigid-type food packaging, such as: pots, containers, tubs, bottles, pouches, and wrapping films [20].

PP is used for the production of cast or biaxially oriented films, whose gas barrier properties have to be improved with coatings or multilayer structures. Sealability is obtained by the use of polyethylene or propylene-ethylene co-polymers, whereas barrier properties are obtained with acrylics or ethylene vinyl alcohol [21]. Such an improvement, accompanied by a barrier against UV light, can also be achieved with metallization (with aluminum) or lamination with aluminum foil [22]. These films can be used for the production of disposable food containers, mainly bags or pouches, as sealed wrappings, as overwraps with meals placed on plastic trays, in cartonboard containers or as lidding on them [19]. Paperboard intended for containers or disposable packages can be coated or laminated with polypropylene films. Such packaging materials can be used for microwave heating of food constituting a ready meal and intended for direct consumption, due to the relatively high melting point of PP [23].

Polypropylene replaced cellophane (regenerated cellulose) films in wrapping confectionery. Its properties, especially crinkle and dead-twist, make it confusingly similar to the counterpart.

Polypropylene and the co-polymer plastics also find extensive use for caps and closures for bottles, pots and containers, and labels [24].

Extensive studies of application safety and exposure have revealed that polypropylene is a completely inert material and does not present a health hazard to the consumer in either handling the plastics or consuming foodstuffs with which PP have come into contact [19,23,25]. Polypropylene packaging materials are recyclable; to indicate this, they contain the internationally recognized symbol of three green arrows in a triangle shape, with the number 5 or PP in the center, which can be found on the bottom of cups, pots, and containers [26].

#### 3.1.2. Polyethylene

Polyethylene (PE) together with the total polypropylene production capacity worldwide amounted to 172 million metric tons in 2017 [27]. According to projections made before the COVID-19 pandemic, in 2023, their global production capacity will account for 230 million metric tons. Polyethylene alone contributes 63% of the global plastic resins demand in 2019, which constitutes c.a. 100 million metric tons [28]. The largest production of PE among other polymers is caused by the reduced weight and increased strength of the products manufactured quickly and inexpensively with different fabrication methods, e.g., plastic welding, compounding, lamination, and extrusion [29].

Polyethylene is produced by polymerization of ethylene alone (homopolymer) or together with other α-olefin monomers, e.g., 1-butene, 1-octene, or 1-hexene (α-olefin co-polymers) applied for the modification of polymer density. Widely used types of polyethylene: Low-Density PolyEthylene (LDPE), High-Density Poly-Ethylene (HDPE) and Linear Low-Density PolyEthylene (LLDPE), together with specialized polyethylene polymers: Very-Low Density PolyEthylene (VLDPE), Medium-Density PolyEthylene (MDPE) and Ultra-High-Molecular-Weight PolyEthylene (UHMWPE) and cross-linked accompanied for polyethylene polymers distinguished on the basis of their density and thus degree of crystallinity, which influences the melting point ranges of the polymers [30].

The last mentioned property is responsible for the particularly important ease with which packaging can be heat-sealed. Polyethylene plastics present good barrier properties to moisture (desired for water-sensitive food product packaging) and only moderate to oxygen (not suitable for easily oxidized food product containers) and organic substances. These properties, together with clarity and stiffness, are strongly affected with the density, crystallinity, molecular weight, and their distribution [31].

Polyethylene susceptibility to oxidative degradation makes it necessary to apply effective antioxidants in the formulations of commercial PE plastics. To prevent the oxidative degradation, phenolic or phosphite types of antioxidants are incorporated to the formulations. Moreover, slip agents (fatty acid amides) and suitable fine particular fillers need to be applied for the improvement of friction and blocking in films made from PE. Another additive for the polymer is antistatic agent (e.g., polyethylene glycol esters), preventing the finished packaging from picking up dirt and handling problems [32].

The main polyethylene forms used for food packaging are films made by cast or oriented processes and bottles or other containers produced by thermoforming and blow molding processes [32].

LDPE and LLDPE, due to their flexible, soft, and stretchable characteristics, are extensively used as flexible films for frozen foods, bakery products, and fresh meat and poultry. The latter polymer, characterized by its crystal-clear transparency, heat-healing strength, and toughness, is frequently applied for cling films. On the other hand, HDPE provides excellent barrier properties for gas and water and is the main polymer used for containers [32,33]. Both types of PE are used in the production of caps and covers for bottles and containers, as well as container labels. Laminate containers made from paperboard coated from both sides with polyethylene films or multi-layer containers made from aluminium, paperboard, and polyethylene film are applied in packaging for takeaway food and beverages [32].

Polyethylene lamination is applied for heat sealing and prevents water vapor, if necessary. In boil-in-the-bag food, polyethylene is combined with polyamide in order to obtain the rigidity of the foil during water boiling [34]. Other combinations of PE are also possible, e.g., with polyethylene terephthalate (PET) or polypropylene (PP), depending on the resulting properties and performance characteristics [35,36]. An additional layer of aluminium foil is incorporated when good barrier properties to oxygen are desired, e.g., in flexible packaging for coffee [37].

In multi-layer packaging materials, consisting of polyethylene, paperboard, and aluminium, PE is applied for its easy sealing and barrier properties to water; the aluminium layer provides a barrier to oxygen and paperboard ensures the required rigidity of the packaging. Such containers are applied as packaging for long-life fruit juices and milk, whereas paperboard coated two-sided with PE is used in the production of coatings for milk products, takeaway foods, and disposable beverage cups. Moreover, PE-coated paperboard is applied for external cartons for many foods in a wide range of temperatures including sub-zero temperatures [32].

The low glass transition temperature (Tg) of LDPE translates to flexibility at low temperatures, which enables the films to be applied for the packaging of frozen food products. LDPE film bags are preferred as simple and effective packaging of food products with a short shelf life and meat or fish products packaged on plastic trays made from foamed polystyrene and overwrapped with polyethylene films [32,38].

Polyethylene, being thermoplastic, is recyclable. In order to facilitate the recognition of PE plastics, internationally recognized symbols are used, identifying them as high-density polyethylene with the number “2” and letters “HDPE” or as low-density polyethylene with the number “4” and letters “LDPE” at the bottom of the packaging [39].

#### 3.1.3. Polyvinyl Chloride

Polyvinyl chloride (PVC), together with polyethylene and polypropylene, is the most widely exploited thermoplastic synthetic polymer. According to estimation, its worldwide consumption was expected to grow from around 47 million metric tons in 2019 to about 51 million metric tons by 2021 [40]. However, the expectations failed, taking into account the global production capacity of 61 million tones reached in 2016 [41]. Continuous growth in PVC production is a result of increasing demand in building and construction, as well as the automotive market and medicine sector due to growing population, urbanization, and increases in income levels [42]. However, the PVC application in packaging is on the second position, between the building and construction and electrical and electronic sectors [43]. PVC is characterized by the ease of processing and blending, high-tensile strength, as well as heat resistant properties, which makes it widely used in a slew of applications nowadays [42].

PVC is produced by a free radical polymerization of vinyl chloride monomer. It can be obtained via block, suspension, or emulsion polymerization methods, leading to powder PVC of different properties. PVC from block process is of the highest purity and can be used for transparent plates with the best dielectric properties. The emulsion process leads to paste-forming polyvinyl chloride intended to create dispersions with plasticizers, with the consistency of gelling pastes at elevated temperatures. This can be applied in coatings of fabrics, paper, or metal surfaces, and for the production of films. PVC from the suspension process is most frequently applied in the building and construction sector for pipes and plates [18].

The low thermal stability, poor impact strength, and relatively high melting point of PVC are responsible for the need of additives application during processing of the polymer. Due to the low thermal stability of PVC (above 160 °C), thermal stabilizers need to be incorporated to the polymer, protecting it from thermal decomposition. Poor impact strength requires impact modifiers, which significantly improve the resistance to sudden shock. Such an additive is not applied in plasticized PVC, which is produced from emulsion PVC by the incorporation of plasticizers (organic esters, e.g., phthalates, adipates, or trimellitates). Other types of additives are lubricants, processing aids, and pigments [44].

PVC is flexible, light, cost-effective, transparent, and tough. It does not affect the taste and look of packaged food and prevents it from contamination with bacteria or fungi during manufacture, distribution, and display, especially in the form of cling film. This, combined with the excellent oxygen and water barrier properties of PVC, provides a longer shelf life and prevents unnecessary waste of food [45].

PVC in packaging is used mainly as rigid film (about 80%), flexible film such as cling film (15%), and closures (3%). PVC provides a very variable and cost-efficient material for the production of packaging for disposable syringes and medical devices, blisters, and presentation trays for a variety of foodstuffs, batteries, electronics, tools and toys, pharmaceutical tablets blisters, toiletries, adhesive tapes, and bottle sleeving, as well as cling film for food [45].

Unplasticized PVC (PVC-U) is applied in packaging for the production of modified-atmosphere extended-shelf-life food trays, general-purpose food trays, and collation or straight-on-shelf display trays. The resistance of PVC to oils together with its toughness and clarity makes it suitable for bottle production, but not for carbonated drinks because of its high permeability to carbon dioxide. Films of plasticized PVC (PVC-P), characterized by stress and cling, are suitable for hand-wrapping of fresh products in supermarkets and domestic usage. PVC is applied in closures of containers and linings of cans in the form called “plastisols”. These are liquids at room temperature, produced from emulsion PVC of fine particle size, which, after pouring on the surface, undergo curing to form rubber-like sealing. Another application in the food area is tubing for the transportation of beer and soft drinks [44].

PVC easily processing together with its advantageous cost-to-performance ratio, good barrier properties, and excellent organoleptic properties, combined with high clarity and visibility of the product, lightweight and good shatter resistance, has effectively replaced glass in packaging. PVC, similarly to PE and PP described above, is recyclable. It can be mechanically treated to form granules (e.g., grinding), that afterwards are melted and remolded into the initial products, or chemically processed (during pyrolysis, hydrolysis, and heating) into its chemical components (NaCl, CaCl_2_, hydrocarbons, and heavy metals) used for the production of new PVC [46]. The international symbol of recyclable products made from PVC contains the letter “3” in a triangle shape [47].

#### 3.1.4. Polyethylene Terephthalate

Polyethylene terephthalate (PET) is the most commonly used thermoplastic polymer resin. In 2020, the worldwide demand for PET reached 27 million metric tons. It is forecasted that by the end of 2030, global PET demand will amount 42 million metric tons [48]. PET is in sixth place, with almost 4 million metric tons of total European plastic converters demand, which constitutes 7.7%. However, taking into account plastic demand in packaging, constituting the biggest part of total plastic demand in Europe with 39.9%, it takes third place in this segment [16].

PET belongs to the group of synthetic polyesters and is produced from terephthalic acid and ethylene glycol in condensation reaction. In the presence of metal catalyst, molecules of both types of monomers react together, releasing a molecule of water. This is followed by a second polymerization stage proceeding in solid state and resulting in the high-molecular-weight polymer [49]. It is crucial for good mechanical properties, providing stiffness, toughness, and creep resistance, while at the same time giving sufficient flexibility to resist bursting and breaking under pressure.

The very high polymer quality required for food is achieved by the application of monomers of the highest chemical purity at the stage of PET synthesis as it is impossible to achieve when the polymer is formed [49]. PET is essentially composed of very-high-molecular-weight species. Little information is known on the migration and toxicity of the low-molecular-weight oligomers, formed due to sunlight-promoted degradation or by interaction with food or beverages [50]. PET itself provides the desired properties for packaging applications; therefore, typical additives for polymers, such as antioxidants, plasticizers, heat or UV stabilizers, are not required. That accounts for greater safety in the use of PET in the packaging industry [51].

The great popularity of PET is a consequence of its glass-like transparency and excellent gas barrier properties enabling carbon dioxide permeation. Moreover, PET is lightweight and offers a favorable toughness-to-weight ratio, which ensures containers up to 1.5 L capacity are shatterproof [49]. PET is a semi-crystalline polymer. After being heated above its glass transition temperature, it changes from a rigid glass-like state into a rubbery elastic form. In this phase, the PET molecules can be stretched and aligned in one direction to form fibers, or in two perpendicular directions to form films and bottles. Sudden cooling down causes the chains frozen with their orientation preserved and formation of PET for bottle-like applications. Continuous heating above Tg of PET causes slow crystallization, giving an opaque, more rigid, and less flexible polymer capable of withstanding moderate oven temperatures. Crystalline PET (CPET) is used for the production of trays and containers intended for heating as well as for the formation of polyester textiles [49,52].

The main packaging applications of PET are containers (bottles, jars and tubes), semi-rigid sheets intended for thermoforming (trays and blisters), and thin oriented films for bags and snack food wrappers [49]. Trays made from PET are destinated for pre-cooked food for re-heating in both microwave or conventional ovens [53]; PET films and metallized foils are used for boil-in-the-bag pre-cooked meals, snack foods, nuts, sweets, long-life confectionery, ice creams, and spreads, whereas PET films with an added oxygen barrier are applied for beer, vacuum-dried dairy products, bag-in-box wine, condiments, coffee, cakes, and syrups [49,54].

PET products, being recyclable, are marked with “01” in a triangle and the letters “PET” on the bottom of the recycling symbol.

#### 3.1.5. Polystyrene

Polystyrene (PS) is the general description of a family of styrene-based polymers, which are used in many areas of industry, from furniture, electrical equipment, and insulation materials to toys, houseware, and packaging [55].

The global polystyrene capacity in 2019 amounted to 15.61 million metric tons and is predicted to grow only very slightly in 5 years’ time [56]. The PS demand volume places the polymer in seventh position with 6.4% of total plastics demand in Europe [16].

Polystyrene is produced in two types: general-purpose polystyrene (GPPS) and high-impact polystyrene (HIPS). GPPS is an amorphous polymer, characterized by high clarity, lack of color, excellent dielectric properties, chemical resistance, hardness, and fragility, which can be easily processed with injection molding extrusion or thermoforming [18,56]. The latter, produced by the polymerization of styrene in the presence of polybutadiene, possess enhanced physical properties and impact strength suitable for food packaging. Moreover, certain types of HIPS are less sensitive to stress cracking [56]. GPPS transparency is lost in HIPS. However, the impact strength appears. Both GPPS and HIPS have poor barrier properties to gases (oxygen and carbon dioxide), which influences the shelf life of food. However, it is not a drawback in the case of yogurt pots, where limited penetration of oxygen is necessary for the fermentation process to be sustained [57].

Another type of polystyrene is expanded polystyrene (EPS), which is used for the production of foamed sheet form. EPS is produced in the simultaneous process of foaming and sintering of polystyrene pellets pumped with pentane. Under the influence of hot steam, the granules increase almost 50 times and join together to form a homogenous and stable material. EPS is used for general protective packaging, sometimes called cushioning packaging and also for packaging for food formed into trays and containers, as well as for disposable beverage cups [58].

Polystyrene can be synthesized by bulk, suspension, emulsion, or solution polymerization of styrene, mainly in the solution or suspension process. The process is exothermic, and a liquid reaction environment enables the temperature to be controlled. The most common method is radical polymerization with the application of organic peroxides as initiators [18,59].

GPPS is applied as containers for a variety of foods and as disposable “plastic glasses” for beverages due to its inherent transparency. Foamed polystyrene is used for the production of trays for meat, poultry, fish, fruit and vegetables, closed containers for eggs and fast foods, and also disposable cups for beverages. These can be additionally coated with a GPPS layer, constituting a barrier between the packaging and food. Thin bi-axially oriented polystyrene films are applied for food packaging carton windows and also as breathable films for over-wrapping fresh products, whereas thick PS films are used for clear vending cups, and tubs for desserts and fruit or vegetable preserves [55]. On the other hand, HIPS is used for the production of pots for dairy products, as vending cups for hot beverages including soups and also as clam-shell packaging for eggs [60].

In spite of numerous applications of PS in packaging, considerable disadvantages need to be taken into consideration. Crystal PS and HIPS sensitivity to high temperature excludes their application in boxes intended for heating in a microwave or oven. Moreover, under the influence of fat present in food (e.g., salad dressing, butter), stress cracking of the PS packaging occurs, followed by decreased barrier function [55].

PS is suitable for recycling. The number “6” with letters “PS” at the bottom of the recycling symbol is reserved for this polymer.

### 3.2. Bio-Based, Biodegradable Polymers

**Figure 4:**
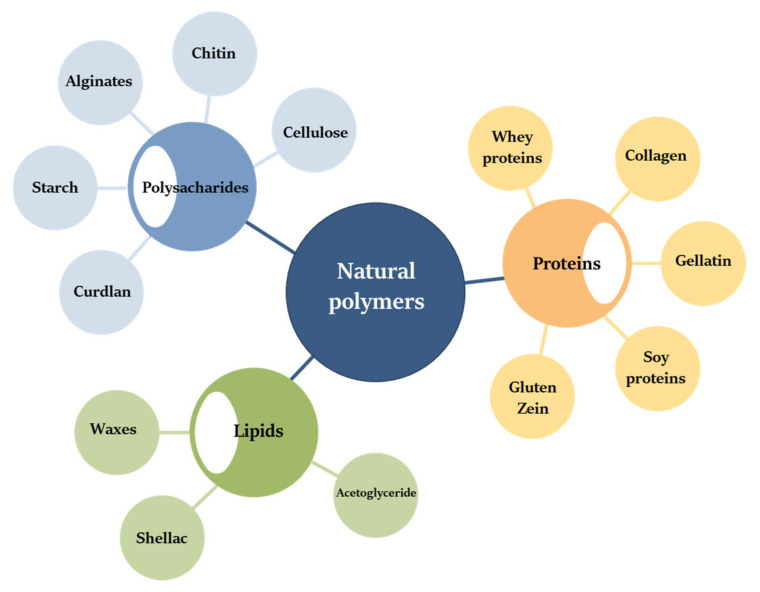
Diagram of natural polymers categories.

Amongst new-generation polymers, which are intensively developed nowadays, both natural and synthetic polymers are found. In general, synthetic and natural polymers are built of repetitive, smaller, regular structures linked by covalent bonds [61]. Regardless of their production, they show similar chemical structures. Replacing conventional plastic polymers with new-generation materials and/or ‘more natural’ counterparts is of interest to both the scientific world and entrepreneurs. Natural polymers can be divided according to their chemical structure: (1) polyesters, (2) proteins, (3) polysaccharides, (4) lipids (Figure 4); or on the base of their origin, for example, polymers of plant origin, animal origin, and microbial origin.

So far, many reviews of the literature on the use of polymers of natural origin, as packaging materials, edible films and coatings, as well as for food shelf-life extension, have been carried out [62,63,64,65,66,67]. Natural polymers are used in the food industry as coatings due to numerous advantages: antimicrobial activity, color preservation, optimized effects on lipid oxidation, improved water vapor permeability, and retained freshness of food for a long time [67,68,69,70]. However, there is no literature review focused on the use of edible biopolymers as raw materials with high potential for the production of disposable dishes, cutlery, cups, and others.

#### 3.2.1. Natural Polymers Produced by Living Organisms

##### Polysaccharides

Cellulose

Cellulose is the most abundant polymer in the natural environment, and can be obtained from rice, wood, cotton, plant biomass, algae, and specific strains of bacteria [71]. It is a linear homopolymer, comprised of glucose units connected by β-(1→4) glycosidic bond. Cellulose extraction is considered to be a difficult process with expensive pre-treatment. Enzymatic hydrolysis with exoglucanases, endoglucanases, and β-glucosidase is used to isolate cellulose from biomass. After purification, cellulose can be chemically modified to cellulose derivatives with improved properties [72,73]. In general, cellulose is water insoluble because of the presence of inter- and intramolecular hydrogen bonding between OH groups, but properties of cellulose depend on the degree of polymerization as well as the source of this biopolymer [74]. Cellulose and its derivatives possess good film-forming properties, which are generally biodegradable, non-toxic, and transparent, with excellent mechanical, thermal, and barrier properties [75,76]. Cellulose, as well as starch, belongs to hydrophilic polymers, being water wettable or swellable and consequently biodegradable; thus, their application in terms of technology is limited. On the other hand, the poor solubility of cellulose is one of the challenges in the commercialization of cellulose in the food industry. Therefore, modifications of these material are still being sought after. Transparent films by industrial cellulose pulp solubilization in tetramethylguanidine-based ionic liquids were investigated by Ribeiro et al. [77]. Carboxymethyl cellulose, an ionic, water-soluble derivative of cellulose, is one of the most promising cellulose derivatives, characterized by good surface properties, mechanical strength, tunable hydrophilicity, viscous properties, availability, and low-cost synthesis process [78]. It is used in various fields such as food, paper, textile, and pharmaceutical industries. However, cellulose acetate, which is used in films and filters, is biodegraded in the environment, but this process is very long [79]. Conjunction of bacterial cellulose (with incorporated yeasts) with carboxymethyl cellulose (CMC) and glycerol to extend the shelf life of packaged food materials were also studied [80]. What is more, the hybrid fiber strategy of long bamboo fibers with short sugarcane fibers was applied in the production of tableware with high tensile strength, superior oil stability, excellent hydrophobicity, and low heavy metal content [81]. Additionally, cellulose with starch can be used to make compostable cups and trays through the hot-pressing method [82].

Bacterial cellulose (BC) is an extracellular polymer of bacteria belonging to *Achromobacter, Alcaligenes, Aerobacter, Agrobacterium, Azotobacter, Komagataeibacter* (formerly *Gluconacetobacter*), *Pseudomonas, Rhizobium, Sarcina, Dickeya,* and *Rhodobacter* genera [83,84]. However, the strains belonging to the genus *Komagataeibacter* are most commonly used in research and the commercial production of bacterial cellulose [85,86]; *Komagataeibacter xylinus* is considered a microbial model in BC production [87,88]. The primary structure of the bacterial cellulose consists of long-chain β-1,4-linked glucose (glucan chains), reaching a degree of polymerization up to 20,000 [89]. Physicochemical properties of BC depend on the specific characteristics of the architecture, its nanostructure, and macrostructure. They therefore depend on both intracellular biosynthesis and extracellular self-assembling [87]. BC is identical in chemical composition to plant cellulose, but characterized by a higher crystallinity, degree of polymerization, purity, water-holding capacity, but also high mechanical strength, elasticity, and shapeability, and gas and liquid permeability [83,90]. It is worth noting that BC was approved by the FDA (the United States Food and Drug Administration) as Generally Recognized As Safe (GRAS) and can be used as a safe food product or food component [91]. The interest in bacterial cellulose as a packaging material is constantly growing, and several studies have demonstrated that BC shows many advantages in its application in food packaging. The latest review article written by Ludwicka and others [92] describes the application of bacterial cellulose in active and intelligent food packaging. Antibacterial activity and higher elastic modulus were obtained by BC impregnation in chitosan solution and described in the study of Kingkaew et al. [93]. The improvement of the tensile strength and barrier performance can be achieved by crosslinking with proteins, which are more easily involved in crosslinking reactions than polysaccharides. The effect of gelatin content on the tensile properties of BC/gelatin composition was studied by Chang et al. [94]. The authors found that a higher gelatin concentration enhances the tensile properties of the composite. On the other hand, the properties of bacterial cellulose can be modified by the addition of other biopolymers (pectin, xylan, gelatin, or carboxymethylcellulose) to the culture medium, which can stimulate the synthesis of BC and enhance its mechanical properties [95,96]. Another approach in the production of composites from bacterial cellulose is the isolation of BC crystalline regions, leading to bacterial cellulose nanocrystals (BCNC) [97]. BCNC with nisin [98] or cinnamon essential oil [99] was successfully used as an antimicrobial composition. What is more, antimicrobial activity and enhanced barrier and tensile properties of films with BCNC were achieved by the incorporation of BCNC into a chitosan dispersion with silver nanoparticles [100]. In general, this strategy can be used for cellulose-based formulations for varied food applications [101]. On the other hand, there are many challenges for the commercial production of BC-based materials for food application [92].

Starch

Starch is a polymeric carbohydrate consisting of two types of molecules: the linear and helical amylose branched amylopectin. Amylose, which is responsible for film-forming properties, is a polymer of α-1,4 anhydroglucose, while amylopectin is a highly branched polymer of short α-1,4 chains linked by α-1,6 glycosidic branching points occurring every 25–30 glucose units [66]. Depending on the plant, starch contains 20–25% of amylose and 75–80% of amylopectin. A higher amylose content results in a greater surface roughness of obtained starch-based materials [102]. Various starch-based products have been developed and commercialized, and conventional processing techniques (extrusion, injection, compression molding, casting, and foaming) as well as novel techniques (reactive extrusion) are used for processing starch-based polymeric materials [103]. Starch is considered as an alternative to plastics derived from petroleum derivatives in the production of packaging films [104]. However, due to their high moisture absorption and poor mechanical properties, starch materials are unstable during processing and storage, and modified starch materials from different plant origins are the subject of numerous studies. Due to the higher lipid content, glutinous rice starch and normal rice starch-based materials are characterized by higher contact angle values than cassava starch [105], while for films containing blackberry pulp, the increased contact angle and lowered surface roughness were observed. Simultaneously, a lower in vitro digestibility rate and higher resistant starch content were noticed. What is more, for films containing blackberry pulp, higher anti-inflammatory activity and higher cell viability were confirmed [106]. Combinations of plasticized starch with protein in order to improve processability and storage properties were examined in the study of Huntrakul et al. [107]. The authors found that pea protein isolate stabilized films during blown extrusion but decreased their flexibility. An increase in the concentration of pea protein decreased the solubility and improved the crystallinity, surface hydrophobicity, and barrier properties against water vapor and oxygen [107]. Films with starch and yerba mate extract were found to be more hydrophobic and tensile resistant [108]. Yerba mate extract and poly(vinyl alcohol) mats were incorporated within potato starch in the study of López-Córdoba [109]. The authors concluded that PVA mat and the yerba extract caused a synergistic effect that increased the elastic module of the biocomposites, while the tensile strength and strain at break were maintained. The results of the study conducted by Righetti et al. (2019) show that starch in the biocomposites of poly(lactic acid) (PLA) with potato acts as filler for PLA and the additional application of biobased and petroleum-based waxes improves the mechanical properties of the composites [110]. In order to improve the mechanical and water-resistance properties of starch bioplastic, epoxidized palm oil or soybean oil can also be used [111]. Improved mechanical properties of obtained polymer were also noted when dolomite filler was introduced into thermoplastic starch, and sonicated dolomite-thermoplastic starch shows better mechanical properties than pristine dolomite [112]. Ren et al. [113] found that sorbitol has a negative effect on the dispersion of the halloysite nanoclay in the starch matrix, but the addition of halloysite improves the mechanical properties for glycerol plasticized system, compared to composites based on sorbitol and glycerol/sorbitol.

Pectins

Pectin forms the most complex class of polysaccharides, composed of heterogeneous groups of glycanogalacturonans and acidic structural polysaccharides. Generally, pectin is a structural acidic heteropolysaccharide of galacturonic acid monomers (70%), a sugar acid derived from galactose [64]. D-galacturonic acid residues are linked at α-1,4 positions, and the acid monomers can be acetylated or methyl esterified. Pectins can be divided into three groups: (1) Homogalacturonans (HGs), Arabinogalactans (AGs), and Rhamnogalacturonans (RGs). Homogalacturonans, the most abundant pectins (up to about 65% of pectins), are homopolymers of α-(1→4)-D-galactopyruronic acid (Galp) methyl esterified units [114]. Arabinogalactans can be distinguished in two groups: AG I (arabino-4-galactans, constituted by a β-(1→4)-Galp backbone with side chains of arabinans) and AG II (arabino-3,6-galactans, constituted by a linear backbone of 1→3 and 1→6-linked galactopyruronic acid units, branched with arabinan chains). Rhamnogalacturonans, known as “the real pectins” are heteropolymers of galactopyruronic acid and rhamnopyranose branched with arabinogalactans chains. Rhamnogalacturonans I have linear a backbone of alternating α-1,4-linked galactopyruronic acid units and α-1,2-rhamnopyranose units, while rhamnogalacturonans II are constituted by a homogalacturonan backbone of about 9–10 methyl esterified galactopyruronic acid monomers [115]. Homogalacturonans are also known as the “smooth region” of pectins, while the rhamnogalacturonans and arabinogalactans are the “hairy regions” of pectins. Pectins are present in all the higher plants and occur in the intercellular or middle lamellar region [116]. Citrus peel, apple pomace, and sugar beet pulp are widely distributed sources of pectin. In the food industry, they are used as stabilizers, thickening and gelling agents, crystallization inhibitors, and encapsulating agents. Pectin gel is formed when homogalacturonans are cross-linked to form a three-dimensional crystalline network in which water is trapped [117]. Coatings from pectin and its derivatives are considered to be used in food-related applications due to their barrier to oxygen, aroma preservation, barrier to oil and good mechanical properties; however, due to their hydrophilic nature, they are not effective against moisture transfer [64]. They are used as coating in fresh and minimally processed fruits and vegetables [118]. It was described that pectin-based coating can enhance the shelf life of lime fruits [118], which can be used for preservation for a short time application [119]. Priyadarshi (2021) found that a 50:50 ratio of pullulan and pectin exhibits the highest thermal stability and surface hydrophobicity, reduced water and oil absorption values, as well as increased strength, at the same time maintaining flexibility and stiffness [120].

Chitosan

N-acetylglucosamine (chitin), a precursor of chitosan, is considered to be the second most abundant biopolymer; however, unlike cellulose, it is mainly found in exoskeletons of crabs, lobsters, crayfish, shrimp, and other crustaceans, as well as in the cell walls of fungi. The form that shows increased solubility in acidic environments is chitosan—partially deacetylated form of chitin. Chitosan consists of β-(1→4)-2-amino-2-deoxy-D-glucose monomers [121] and can be possessed from different sources: crustacean shell waste (20–30%), *Nephrops* spp. and *Homarus* spp. (60–75%). Methods of chitosan preparation include three stages: (1) removing calcium carbonate from the shell (demineralization), (2) removing protein and organic compounds other than chitin (deproteinization), and (3) converting chitin to chitosan (deacetylation). This polymer is characterized by many functional properties; on the other hand, a major limiting factor is its poor solubility, which would enable wider industrial application. Chitosan can be modified by physical or chemical processes such as grafting, cross-linking, and substituent incorporation [122]. Modified forms of chitosan, such as phenolic acid-grafted-chitosan, exhibit enhanced antioxidant, antimicrobial, antitumor, anti-allergic, anti-inflammatory, and anti-diabetic activities [123]. PLA/chitosan composite film is an interesting alternative to plastics [124]. Chitosan and chitosan derivatives show antimicrobial activity with high potential within a number of industries [124]. Chitosan incorporated with extracts of propolis, mango leaf, thermoplastic maize starch, silver nanoparticles, and tea polyphenols show antimicrobial activity against Gram-positive and Gram-negative bacteria as well as against molds and yeasts [125,126,127]. Interestingly, the incorporation of tea polyphenols together with silver nanoparticles cause an improvement in the mechanical properties of the obtained composite, as well as in a higher antioxidant resulted [128]. More importantly, it is considered as sustainable, environment friendly, alternative to synthetic packaging materials, with gas and aroma barrier properties, as well as increased shelf life of the products.

Sulfated Polysaccharides

Sulfated polysaccharides (SPs) are present in the cell wall of marine algae or seaweeds constituted mostly of cellulose and hemicellulose with high carbohydrate content but low calories and fat content. Due to the cross-linkage of sulfate group ions with complex molecules of polysaccharides, the molecules of SPs are negatively charged [129]. Fucoidans (from brown algae), carrageenans (from red seaweeds), ulvans (from green seaweeds) are main SPs.

Fucoidans

Fucoidans are a long-chain SP found in various species of brown algae: *Stoechospermum marginatum, Sargassum (S. ilicifolium, S. marginatum, S. marginatum, S. myriocystum, S. wightii*, which yields 71.5 mg of fucoidan from 1 g of seaweed dry weight), *Dictyota dichotoma, Turbinaria (T. conoides, T. decurrens, T. ornate*). The main sugar found in the polymer is fucose, while other sugars are galactose, xylose, arabinose, and rhamnose. Fucoidan is composed of two chain structures: one with (1→3)-α-L-fucopyranose as the chain and the second with α-L-fucopyranose linked by (1→3) and (1→4) bonds. Sulphate groups at the C-2 or C-4 of both skeletons can occur [130]. In general, the structure of fucoidans is dependent inter alia on seaweed species; for example, fucoidan from *Fucus vesiculosus* is composed of fucose and sulfate, whereas *Padina pavonia* contain fucoidan constituted with fucose, sulfate, xylose, mannose, glucose, and galactose [131]. Biological activities of the polymer include antitumor, antioxidant, anticoagulant, antithrombotic, immunoregulatory, antiviral, and anti-inflammatory effects [130], while functional properties include gelling, chemical reactivity, improving quality, and controlling moisture [129,132].

Carrageenans

Carrageenans are natural polysaccharides obtained by extraction from seaweed containing large amounts of sulfur, which is closed in the form of sulphate groups in the structure of the plant. The red algae of the *Rhodophyceae* family are most often used for this purpose [133]. A high level of carrageenan is obtained from *Kappaphycus alvarezii*. Carrageenan contains 15–40% of ester-sulfate. Units of 3,6-anhydrous-galactose (3,6-AG) and D-galactose are linked by α-1,3 and β-1,4-glycosidic bond forming carrageenan [134]. From a chemical point of view, there are many isomers, but three main forms have been used in particular: kappa, lambda, and iota [135]. Carrageenan acids are unstable in their pure form; therefore, only the salts of these acids have found industrial application. The most commonly used salts are calcium, sodium, and potassium. Not all isomers are able to react with specific ions, e.g., the lambda isomers do not form gels by reaction with ions. It is believed that mixtures of individual isomers are the most promising. For example, the combination of two kappa isomers with one iota resulted in a gel of high elasticity. The attractiveness of carrageenan is based on its gelling properties, but it has no nutritional benefits. The gel strength, solubility, and temperature stability are affected by the level of ester sulfate, and increased ester sulfate level lowers the mechanical property of SP. They are used in the production of edible packaging, film coatings, and blends, and the addition of starch improves the mechanical strength, gelling strength, and barrier properties [129]. Carrageenans show great potential as an ingredient in gradual-release drugs. Hydrogels obtained as a combination of carrageenans and alginates can be used in targeted drug delivery [136]. What is more, they can be applied in milk products and dietetic formulations, but >2% of carrageenan in food products results in adverse health effects and degraded carrageenan is prohibited as it causes cancer [137].

Ulvans

Ulvans are a polyanionic heteropolysaccharide constituted by β-(1–4)-xyloglucan, glucuronan, and cellulose in a linear arrangement, occurring in green algae *Ulva* (*U. conglobate* and *U. prolifera*) [138]. The ulvan content varies from 2.7% in *U. flexuosa* to 40% in *U. armoricana* [139]. Ulvan can be applied in food, pharmaceutical, and biomedical products [140]. The biological activity of ulvan as antioxidant and antimicrobial activity against human, plant, and animal pathogens were demonstrated in the study of Amin [141]. Ulvan-based gels, fibers, films, nanomaterials, and composites arouse more and more interest [142]. Morelli et al. (2019) obtained ulvan-based emulsions with promising properties as a stabilizing agent for food and cosmetic application [143]. Shalaby and Amin (2019) found that a 1–2% addition of ulvan polysaccharides stimulated the growth and activity of probiotic bacteria [144]. The potential of ulvan as a carrier of antimicrobial agent (nisin) against Gram-positive bacteria was evaluated by Gruskiene [145]. What is more, Guidara and others (2019) found that ulvan can be used as a film layer forming system, showing solubility, barrier, optical, and good mechanical properties, which are important for food and packaging products [146]. Ulvan-based film with glycerol was also obtained by Ganesan et al. (2018) and improved physicochemical and mechanical properties with decreasing water vapor permeability were noted [147]. Active films based on ulvan with glycerol or sorbitol as a plasticizer were studied by Guidara and co-workers (2019). The authors found that enzymatic–chemical extraction results in more beneficial impacts on the optical, thermal, structural properties, and glycerol results in the compact structure of films, lower temperature of transition, and greater antioxidant property of the obtained films [146]. It is believed that ulvan functions have broad potential, but further research into this polymer is required [148].

Alginates

In terms of chemical structure, alginates are mannuronic and guluronic acid polymers can be obtained from specific species of algae, mainly *Ascophyllum nodosum, Laminaria hyperborean*, and *Macrocystis pyrifera*. Alginic acids, as these compounds are referred to, are chemically converted into calcium or sodium salts, since only in this form do they exhibit favorable properties. Salts made with monovalent cations, such as sodium, are liquids that exhibit high viscosity [149], while bivalent cations, such as calcium, result in gel structure [150]. The structure and properties of the resulting product are also influenced by ratio of the number of individual acid units, which defines its further properties, e.g., flexibility. The higher content of guluronic acid in the structure will ensure a higher concentration of Ca^2+^ ions, which affect higher gel rigidity. On the other hand, if they are present in smaller amounts, the gel will be softer and more flexible. Due to its antimicrobial, antioxidant, and immunostimulatory abilities, alginates are widely used in the food and beverage industry as well as in the biomedical industries [151,152].

Curdlan

Curdlan is a polysaccharide formed from glucose monomers linked by beta bonds between the first and third carbon of successive monomers. It belongs to the compounds that are soluble in alkaline solutions with a pH above 12; it is insoluble in water and other organic solvents such as methanol and ethanol. Curdlan is produced by bacteria belonging to the genus *Agrobacterium* (former taxonomy: *Alcaligenes faecalis* var. *myxogenes*) [153,154]. It is believed that the synthesis of the compound depends on the environmental stressors. Bacteria are able to synthesize it from various carbon sources, including glucose, maltose, fructose, and sucrose. According to Wu et al. (2018), 2% glucose, maltose, and sucrose as the carbon source showed better curdlan production than 2% galactose or fructose [155]. Similar results were obtained by Lee’s team, where the highest amount of curdlan was obtained in the medium with 10% maltose [156]. An interesting ability of a polysaccharide is to change its elasticity as a gel under the influence of temperature. As a result of heating, it gains considerable strength. The fact that it has no taste, smell, or color speaks for its use in food. It also does not require additional chemical transformations, such as alginates [154].

Agar, fucoidan, carrageenan, ulvan, and others can be used for both edible as well as non-edible film or wraps, bags, and covers with enhanced barrier properties. What is more, these natural polymers can be blended with other polymers (polylactic acid, polyolefins, polyhydroxy butyrate) as well as with nanoparticles and nanocrystals [157]. Due to their biodegradability and low environmental impact, they are an interesting alternative to synthetic polymers used in the production of single-use plastic materials.

##### Proteins

Zein

Zein is a prolamin predominantly present in the endosperm. Zein may be obtained from corn or corn byproduct from the production of ethanol, starch, or oil [158]. More than 50% of amino acids in zein are hydrophobic (leucine (20%), proline (10%), and alanine (10%)), while the main hydrophilic amino acid is glutamine, with 21–26% [159]. Based on different solubility and molar mass, three fractions of zein are listed: (1) α-zein (21–25 kDa), defined as prolamine of corn, obtained in greater quantities in the commercial (80% or prolamine); (2) β-zein (14–24 kDa)—10–15% of prolamine; (3) γ-zein is obtained in the content of 5–10% [160]. Generally, zein has negligible content in lysine and tryptophan, which, together with its poor solubility in water, limits its use for human consumption. On the other hand, zein is known as an alcohol-soluble protein. Due to its poor solubility in water, zein is applied for coatings production and the obtained films are characterized by the water vapor barrier property. However, without plasticizer, zein-based materials are characterized by very brittle structure, moderate moisture barrier, oxygen barrier, and mechanical properties, which results in extremely limited application in the food industry [161,162]. Zein-based materials for the food industry are obtained through extruders provided with slit dies, where additives such as oleic acid can be used. Zein was used for the preservation of tomatoes [163] and fruits, showing a reduction in weight loss [164], as well as wrap for food packaging applications to the protection of fresh-cut fruits and vegetables from dust [165]; active zein-based films were used as antimicrobials against *Listeria monocytogenes* and to prevent lipid oxidation in fresh cheese [166]. The protein was also investigated in the production of packaging materials. Modification of zein with oleic and linoleic acids as plasticizers increased the elongation percent and decreased the water absorption of the obtained film [167]. Zein-based films plasticized by polyols (sorbitol, glycerol, and mannitol) were investigated by Ghanbarzadeh and others [168]. Bioactive packaging films with zein incorporated with orange-peel oil were proposed as packaging that ensure the safety of food products [169]. Phenolic compounds such as gallic acid, *p*-hydroxy benzoic acid, ferulic acids, catechin, flavone, and quercetin were used as antioxidant and antibacterial additives to zein-based films [170]. EDTA, lauric acid, and nisin, as well as their combinations were incorporated into the zein film as antibacterial agents, for potential application in the food industry [171].

Gluten

Wheat gluten (WG) obtained as an agricultural byproduct is considered as a promising material in the production of packaging materials. WG consists of insoluble gliadins (28–55 kDa) containing small numbers of disulfide and sulfhydryl groups; and water-soluble glutenins (500–1000 kDa), which link together through intermolecular covalent disulfide bonds [172]. In the presence of hydrophilic plasticizers, WG can be easily processed by extrusion at 60 °C [173]. WG is characterized by viscoelastic properties—plasticized with glycerol, gluten forms structured viscoelastic solid with pseudo-plastic behavior [174]. High pressure and temperature cause significant WG strengthening, and gluten proteins show greater gel strengthening than the smaller soy proteins [175,176]. What is more, its thermoplastic properties as well as high capacity for chemical modification offer the possibility to develop a range of materials [177]. These proteins can be combined with different additives, through different processes such as casting, as well as thermomechanical methods (compression molding, extrusion), which make it possible to obtain a variety of products [178]. Extrusion results in a greater gluten compatibility with plasticizer compared to compression, and tensile strength is enhanced at pH 9 [179]. Reactive extrusion with chrome octanoate as catalyst was used in the production of gluten/poly(ε-caprolactone) food packaging films [180], and the films were recommended as potential shape memory food packaging materials. The effect of sucrose and trehalose in gluten-based bioplastics was evaluated by Alonso-González et al. [178]. The authors found that sugars can act as a filler or plasticizer, and it depends on the presence of water [178]. Nanocomposite-based packaging film comprised of gluten modified with carboxylated cellulose nanocrystals [181], and the effect of clay nanoparticles on the biodegradability of wheat gluten-based materials was studied [182]. WG with lipids (beeswax, stearic and palmitic acids) was applied as a film and coating on refrigerated strawberries [183]. An increased hydrophobicity of gluten-based material was found by the addition of epoxidized soybean oil [184]. Wheat gluten was also used in the production of papers for food packaging [185]. Increased antioxidant activities and decreased oxidation of sesame oil were noted after the application of WG/chlorophyll films [186]. Antimicrobial and antioxidant activities were achieved through the application of thyme oil to gluten-based edible films [187].

Soy Proteins

Soya as well as pea and peanut proteins consist of two main fractions: small (10–20 kDa) and water-soluble albumins (up to 20%); and globulins—in soya, these are β-conglycinin (trimer, each unit 52–72 kDa) and glycinin, which is a hexamer with six subgroups, and each subgroup is consisted of an acidic (~35 kDa) and a basic (~20 kDa) polypeptide linked together by a disulphide bond. Soya proteins are characterized by gelling properties, and gels from β-conglycinin are soft and rather elastic, whereas gels obtained from glycinin are harder [188]. What is more, soy proteins show good film-forming properties with potential application as films and coatings for food application, and the parameters of the obtained materials can be improved by the addition of various plasticizers or production processes [189,190]. Despite the fact that glycerol is the most common [191], other plasticizers are used for the better properties of the obtained materials: propylene glycol, polyethylene glycol, sucrose [192], sorbitol [193], acetic anhydride, succinic anhydride, calcium cations, and formaldehyde [194].

Collagen/Gelatin

Gelatin, a byproduct of an animal slaughtering and processing, is derived from the fibrous insoluble protein collagen. Gelatin is a mixture α-chains, β-chains, and γ-chains, which are composed approximately of 50% carbon, 7% hydrogen, 17% nitrogen, and 25% oxygen, with a typical amino acid composition of Gly-Pro-Arg-Gy-Glu-4Hyp-Gly-Pro [195]. Type A gelatin (pigskin gelatin) is obtained from acid-treated collagen, whereas type B gelatin (beef skin gelatin) is derived from an alkali-treated precursor [195]. Properties of gelatin can be divided into two groups (1) associated to surface behavior (protective colloid function, emulsion and foam formation and stabilization, adhesion and cohesion, and film-forming capacity) and (2) those related to gelling (thickening, texturizing, and water-binding capacity, gel formation) [196]. Gelatin films from different sources (fish versus poultry) show different properties, which can be a result of different amino acid compositions, particularly glycine, proline, and hydroxyproline [197]. For example, gelatin-based films from pollock show lower water vapor and oxygen permeability than films from mammalian-derived gelatin [198]. Films obtained from protein are generally more resistant to solvents compared to polysaccharides, and the increased concentration of polymer improved the mechanical properties and water and oxygen permeabilities [199]. Generally, collagen is usually used with other biopolymers such as agar or alginate with incorporated silver nanoparticles [200], casein, keratin, soya proteins [201], starch [202], rice bran protein [203], and zein protein [204,205]. In general, crosslinkers, strengthening agents, plasticizers, or other additives with antimicrobial or antioxidant properties are commonly applied to improve functional properties of gelatin-based materials [196,206]. Improvement of gelatin-based material can be obtained by the addition of chitosan [207], shellac [208], κ-carrageenan [209], or saponins [210]. Gelatin-based films with organic fillers and nanometals such as nanosilver particles, nanocopper particles, zinc oxide nanoparticles, or titanium dioxide nanoparticles show strong antimicrobial and antioxidant activities, as well as preventing UV light transmission [211,212,213,214]. Natural compounds with antimicrobial and antioxidant activities can be added to gelatin films: oregano, rosemary, and leaves of murta [197], as well as astaxanthin [215]. Antimicrobial potential against pathogens of the materials used with collagen/sodium alginate/sorbitol, collagen/sodium alginate/glycerol, tapioca starch/sodium alginate/glycerol, and predatory bacterium *Bdellovibrio bacteriovorus* were also evaluated [216]. What is more, natural additives such as anthocyanins not only increase the antioxidant activity of the obtained gelatin-based films but also influence their mechanical and water resistance [217].

Whey Proteins

Whey is known to be a byproduct of milk production, containing various proteins in the form of protein concentrate (WPC) or protein isolate (WPI). The content of proteins in these products are different; WPI have at least 90% of proteins [218], while the concentration of proteins in WPC ranges from 34% to 89%. The main constituents of whey proteins include α-lactalbumin, β-lactoglobulin, bovine serum albumin, immunoglobulins, and bovine lactoferrin [219]. Whey-based films are usually obtained by casting and drying aqueous whey protein isolate [220]. The materials obtained from whey proteins are characterized by favorable properties (transparent, elastic, odorless). The plasticized whey-based films can be obtained through heating [221], while the improvement of the materials can be obtained through the application of physical (ultraviolet radiation, ultrasounds) and chemical methods (alkalization) [161]. Due to the fact that whey is a hydrophilic protein, the materials obtained have a moderate moisture barrier, as a result of which the water vapor permeability of films obtained from these proteins is high [222]. The barrier properties of the resulting films can be improved by adding materials of a hydrophobic nature, such as essential oils: tarragon [220], cinnamon, and rosemary [223]. In addition to glycerol, the hydrophobicity of whey-based films can also be improved by the application of plant oils, waxes, and fatty acids [224,225,226]. Lower water sorption can be also obtained by the addition of polyvinyl alcohol [227], while mechanical resistance and water vapor barrier can be improved by the application of colloidal nano-silica [228]. Antimicrobial and antioxidant activity of the whey-based materials can be improved by the incorporation of water-soluble chitosan [229], chitosan nanoparticles with rosemary or cinnamon extracts [230], lysozyme with polyacrylic acid [231], or *Fucus vesiculosus* L. extract [232].

Lipids and Waxes

The application of oils and fats, waxes in coatings, and edible packaging material, to improve the parameters of obtained materials (e.g., reduce water vapor permeability), is an area of interest for numerous studies [233,234,235,236]. Waxes, characterized by high-molecular masses, are both of plant and animal origin. They are composed of hydrocarbons (1–60%), esters (5–20%), free alcohols (4–50%), fatty acids (10%), aldehydes, and ketones. Natural waxes inhibit the life processes of plants after harvesting; thus, waxes contribute to their longer shelf life [237]. Due to the fact that their presence causes a decrease in the humidity loss and evaporation, their applications as additives to edible materials is gaining attention [238]. Similar to waxes, lipids used in the production of edible coatings and films are of plant and animal origin. Coatings from lipids are shiny and lose less humidity; they also reduce the cost and complexity of packaging. In general, wax and lipid-based films are characterized by weaker cohesion, flexibility, and gas-barrier properties than protein- and carbohydrate-based materials [222]. Thus, unlike proteins and polysaccharides, lipids are not able to form cohesive films and cannot be used as edible films alone [239]. Consequently, lipids are used as additives that provide a more hydrophobic nature of the obtained materials, introducing increased water barrier properties. Another category in lipid-like packaging, essential oils provide strong antimicrobial activity against pathogenic and spoilage microflora [240]. The group of fats, oils, waxes, and essential oils is relatively diverse in terms of structure, and thus functions that they play in edible packaging used in food production.

Polyhydroxyalkanoates (PHAs)

Polyhydroxyalkanoates (PHAs) are biopolymers belonging to the polyester group. Their monomers are hydroxy acids, which can form chains of several to tens of thousands of units. Due to such a different length of the PHA polymer, separate names were defined for those composed of several units (scl-PHA), a dozen (mcl-PHA) as well as for long chains (lcl-PHA). PHAs belong to natural polymers because they are produced by mainly Gram-negative bacteria. Based on the observation of the bacteria *Cupriavidus necator*, *Chromatium vinosum*, and *Pseudomonas aeruginosa*, an interesting relationship between the growth environment and production of some PHAs was noted [241]. The biosynthetic pathway of PHAs is related to the Krebs cycle, beta oxidation, and the synthesis of fatty acids. In these bacteria, the production of PHA in rich and poor environment was investigated. In the enriched media, the production of large amounts of coenzyme A, which inhibits 3-ketothiolase (the enzyme essential in the PHA production pathway) was found. Therefore, the production of much lower amounts of PHA was observed [242]. On the other hand, in a nutrient-limited environment, there is no such high demand for energy and growth, and the level of coenzyme A is at a lower concentration, allowing acetyl-CoA to be diverted directly into the PHA production pathway [243]. The microorganism used for the industrial production of PHA is *Ralstonia eutropha*, which shows the ability to degrade chloroaromatic compounds and other chemical pollutants. It can accumulate large amounts of PHA, producing it autotrophically, using a wide range of compounds as a carbon source, including alcohols, organic acids, vegetable oils, sugars, and also those that are post-production waste of the food industry [244]. PHAs in the form of composites, nanocomposites, multilayer films, paper coatings, and active food packaging, with particular emphasis on the potential of such materials for food packaging applications, were intensively discussed by Masood [245].

#### 3.2.2. Synthetically Produced Polymers

##### Polylactide (PLA)

Polylactic acid (PLA) is a biodegradable polymer obtained by the polymerization of lactide obtained from L(+)- or D(−)-lactic acid (2-hydroxypropionic acid) produced by fermentation or chemical synthesis. In the last decade, the demand for PLA has experienced a massive boost, and it is one of the most wanted materials for many industrial applications. According to Jem and Tan [246], the PLA world production in 2019 was estimated to be around 190,000 tons, whereas Ncube et al. [247] assessed that it should exceed 300,000 tons by 2024.

Lactic acid can be produced by fermentation of a wide spectrum of raw materials from pure sugars, through low-cost, starch-rich, and lignocellulose-based feedstocks to byproducts from the food industry or even municipal wastes. To obtain lactic acid from renewable resources, the monocultures of bacteria (such as lactic acid bacteria, *Bacillus coagulans*), molds (e.g., *Rhizopus* sp., *Aspergillus* sp.) or genetically modified microorganisms as well as microbial consortia were successfully employed. However, some bottlenecks are present during the biological production process of lactic acid; they are related to low fermentation yields along with unsatisfying yields and the high cost of lactic acid separation and purification [248,249,250]. Production of lactic acid via biological means requires the optimalization of the composition of the fermentation medium (elimination or reduction of expensive additives), as well as the selection of appropriate process parameters that are closely dependent on the selected lactic-acid-producing microorganisms [251].

PLA properties differ depending on their chemical composition—the relationship and distribution of L-, and D-stereoisomers, and comonomers. PLA purity (crystallinity, and enantiomer proportion) impacts mechanical, thermal, and barrier (from water and gases) properties [251]. It was highlighted in many studies that even a small change in the ratio of two stereoisomers of lactic acid impacts the barrier properties of PLA. This polymer has a long tradition of use in pharmaceutical and chemical applications. Nowadays, it is produced mostly as bio-based material for applications in food packaging (for disposable tableware, bottles, containers, and foils), medical applications, and textiles. PLA, PEF (polyethylene furanoate), and PHA (polyhydroxyalkanoate) derived from plant-based feedstock are considered as modern materials with comparable or even better properties over traditional polymers such as PET or PS [10,251].

PLA is a transparent, biodegradable, compostable, hydrophobic, and biocompatible polymer. Due to its thermoplastic properties (commonly improved by the application of food-grade plasticizers), it can be processed into films, sheets, and molded products by thermoforming, blow- or injection molding, extrusion, and film blowing or stretching [247]. PLA is popular for the production not only of shopping bags but also for cups, trays, films, containers, bottles, wrapping, stirrers, all types of cutlery, straws, and foams.

PLA has attracted a lot of attention as an excellent raw material for the preparation of composites combined with a long list of biodegradable and non-biodegradable materials. Blending PLA with other materials helps to increase the availability of low-cost products with improved thermal, mechanical, and optical properties into the market. PLA was blended, for example, with such natural fibers as flax, hemp, kenaf, jute, abaca, pineapple, coir, cotton, and banana. PLA was also found to be blended with nanocellulose, PBAT, PCL, PBS, PE, LLDPE, PS, PET, PP, and PVC [10,247,252]. As for many other polymers, PLA and its composites may be modified by coupling with antimicrobials of natural and synthetic origin to obtain innovative packaging materials [10].

##### Polyethylene Furanoate (PEF)

Polyethylene furanoate (PEF) is a newly developed bioplastic originated from renewable resources. Its global market size was valued at USD 27.1 million in 2019 and is expected to expand and achieve USD 44.5 million in 2027 [253]. It is a promising material considered as an alternative to fossil-fuel-based polyethylene terephthalate (PET) [254]. It is mainly used for the formulation of bottles, films, and fibers [253]. PEF can be fully recyclable; however, currently, it is not biodegradable. PEF has several advantages over PET—lower melting temperature (more than a 100 °C), higher glass transition temperature T_g_, and twice as high moisture barrier properties [254].

This polymer can be synthesized by polycondensation, or much quicker by ring-opening polymerization (ROP) from cyclic PEF oligomers. It is polymerized from 2,5-furandicarboxylic acid (FDCA) and monoethylene glycol (MEG). PEF was preliminary considered as safe for food contact, due to a fact that the migration of the furan-2,5-dicarboxylic acid did not exceed 5 mg/kg food [255].

##### Polybutylene Succinate (PBS) and Polybutylene Succinate Adipate (PBSA)

Global production of polybutylene succinate (PBS) has increased ca. 10 times during the last decade. From 100,000 tons in 2013, it reached 1 million tons in 2020. Such an increase has not been observed in the case of other biobased polymers, including polylactide acid (PLA). PBS is nowadays produced exclusively in Asia. Its production is expected to grow due to the low cost and availability of succinic acid [256].

PBS is a thermoplastic polymer resin belonging to the polyester family. This biopolymer is produced in the condensation between succinic acid and 1,4-butanediol [257] trans-esterification reactions [258]. PBS from the latter process is characterized by the best set of final properties such as balanced mechanical properties, excellent biodegradability and thermoplastic processability [258]. Polybutylene succinate adipate (PBSA) is a copolymer of PBS and polybutylene adipate (PBA) with properties strongly depending on the copolymer composition [259]. The properties of PBS and PBSA are similar to polypropylene [257] and polyethylene [10].

High resistance and compatibility with fibers make PBS suitable for packaging, biomedical, and agriculture industries [258]. Polybutylene succinate is extensively used in food packaging, e.g., disposable tableware and paper cups, due to its excellent gas barrier properties. According to the Biodegradable Products Institute, PBS has been certified as compostable and is available for food contact grades. For this reason, it is dedicated for single-use food packaging and domestic compostable end-of-life products, which cause the continuous growth of PBS production in forecast [258].

PBS is a semi-crystalline polymer with an ester group in its chemical structure, being sensitive and prone to degradation under the influence of water [260]. It occurs in two crystal polymorphs, α and β. Polybutylene succinate is characterized by a low melting point (not exceeding 115 °C), depending on the product, which enables its easy and inexpensive processing [261]. PBS can be processed in a variety of ways, including extrusion, injection molding, and thermoforming. PBS itself is rather brittle and rigid, which can be omitted by mixing or blending with other polymers and materials. PBS has excellent thermal stability and mechanical properties. However, it is deprived of gas barrier properties and softness [260].

PBS has good compatibility with natural fibers such as curaua or jute. The incorporation of curaua fibers increased the impact strength and flexural strength (by the 64%) as compared with neat polymer [262]. On the other hand, the jute fibers improved the tensile strength by ca. 520% and tensile modulus by ca. 3500% in comparison to PBS alone. The best properties were obtained when 50% jute material was applied [263]. Due to the presence of natural plant fibers, containing hydroxyl groups, the water absorption improves as a consequence of introduced hydrophilicity [264].

PBS easily degrades in soil in comparison with synthetic polymers. It can be degraded by fungi and bacteria under natural conditions [265]. Granulated PBS loses almost 80% of its mass after 90 days, whereas the loss of powdered or film PBS is even higher [266]. PBS can decompose even in sophisticated conditions, e.g., under the influence of CO_2_ or lipase solution [267]. The content of natural components (e.g., fibers) increases at the pace of decomposition by at least twofold [268].

Due to the outstanding biodegradability and biocompatibility of PBS, it has risen in popularity as a viable substitute for synthetic packaging. Its high transparency and rigidity make PBS suitable in many applications (from agriculture to civil engineering) and in packaging, especially for mulching films, compostable bags, nonwoven sheets and textiles, catering goods, and foams [260,269]. Puchalski and colleagues [259] confirmed that PBSA is more susceptible for biodegradation than PBS, during an experiment on their biodegradation in soil, composting, and by artificial weathering.

## 4. Dishes Made of Wastes from the Agro-Food Industry

Waste from the agro-food sector is generated in large amounts, mainly in agricultural, horticultural, and livestock farms; sugar factories; distilleries; and other food production and processing plants. Depending on the industry, waste management practices are different. A large proportion of them are pomace and bran. For example, during the production of juices and beverages, pomace constitutes the main waste mass in the amount of up to 25% of the raw material used. They have valuable properties, including powerful antioxidant activity [270]. Currently, pomace is used mainly as a component of feed, as they are a source of dietary fiber, but new directions for their management are still being sought. Different agro-food byproducts are gaining more and more interest such as sources of natural food additives [271]. Wheat brans are a byproduct of the wheat milling process and, like pomace, are used as animal feed. In turn, oat brans are sources of dietary fibers and oat bran extract is also a natural emulsifier [272]. Agri-food waste is more and more often used for the production of biodegradable cups, plates, different tableware, and 3D objects. The dominant among them are products obtained from brans of various cereals such as rye, barley, oat, wheat, and buckwheat, or from apple pomace or pineapple and orange peels [273,274].

Apple pomace consists of low protein and high sugar, mainly cellulose, starch, pectin, and insoluble lignin. These substances can be used for bioplastic production. 3D objects from apple pomace were prepared by using solution casting and compression molding techniques [273].

On the other hand, the production of biodegradable disposable paper cups with acceptable strength properties, used pineapple peels, orange peels, and Mauritian hemp [274]. In the production of cups, soda pulping followed by vacuum molding has been used.

Areca palm (*Areca catechu* L.), sal tree (*Shorea robusta* Gaertn.), Maloo creeper (*Bauhinia vahlii* Villar), banana (*Musa acuminata* Colla), and coconut tree (*Cocos nucifera* L.) byproducts may also serve as raw materials for preparation of biodegradable takeaway containers. This feedstock is traditionally used in Asian countries to serve meals, and according to Gautam and Caetano [275], they are promising to be used more broadly as an alternative to plastic.

In recent years, interest in the production of biodegradable and compostable tableware has been increasing more and more, which can be observed by browsing the databases. On the Web of Science pages, by entering the phrase biodegradable tableware in 2012–2019, there are only one to two records, while in 2020 alone, there are already five hits.

Among the descriptions of the use of byproducts for tableware production, an article by Olt et al. [276] is noteworthy. The authors present the possibilities of using wheat, rye, and buckwheat brans and their mixtures to the plate production. They also note that to reduce the cost of production, the recipe may include ground cereal straws and chaff. The authors also emphasize that in the production technology, attention should be paid to the surface treatment of tableware. The interest in biodegradable tableware produced from bran and other byproducts from the agro-food industry in the world took place much earlier. The first patents were created at the beginning of the 21st century, but more and more have been registered in the last decade [277,278,279,280]. Numerous popular science publications that describe the interest in this area and present local products made of biodegradable and compostable materials can be found on the Internet.

One of the leading producers on the Polish market, but also operating on an international scale, is the company Biotrem [281], which produces disposable bran dishes. They are suitable for serving hot and cold dishes and can also be used in ovens and microwave ovens.

PriestmanGoode presented the “in-flight tray concept” designed as part of the studio’s “Get Onboard” project [282,283]. The proposition includes a reusable tray made from coffee grounds and husks blended with a lignin binder. The tray can be used along with reusable base dishes made from wheat bran. In detail, a side dish is covered with a lid made from algae or banana leaf, and an edible dessert dish has a lid made from wafer. Small capsules used for sauces are made with seaweed. In the set, a reusable cup with an exterior made from rice husk, PLA binder, and algae lining is proposed. The dish designed for a hot main meal is covered with a bamboo lid. Passengers may use a reusable coconut wood spork.

## 5. Edible Tableware and Cutlery—Strength and Microbiological Safety

The next step in replacing plastic serving products is to design and use products that are not only biodegradable and compostable but also edible [284]. According to the report “The Edible Cutlery Market by Product, Raw Material and Application: Global Opportunity Analysis and Industry Forecast, 2019–2026” offered by ResearchAndMarkets.com (accessed on 17 July 2021) the global edible cutlery market was estimated at USD 24,860 thousand in 2018 and is expected to reach USD 56,970 thousand by 2026 [285].

Some edible food packages have been well known for many years. Examples of this type of “packaging” are waffle ice cream cones, as well as dry and sweet cake-made baked bowls, or chocolate cubs and bowls, ice bowls, and drinkware, or simply hollowed out fruit and vegetables or bread. Their main advantage is that they are eaten together with the meal, so the problem of their utilization practically does not exist. Intensive work is currently underway, both on the part of economic subjects, research and development centers, and scientists on the creation of new solutions in this field. The edible tableware and cutlery market enjoys increasing interest every year, as more and more people understand and try to implement the zero-waste trend. Despite this, the consumer habits are difficult to change in a wide range. The main producers of large amounts of undeveloped waste are Asian countries such as China, India, Indonesia, Philippines, Bangladesh, Thailand, Vietnam, and Sri Lanka [286]. However, this problem is also not solved in Europe or America. Although only single scientific articles on the production and use of edible tableware are available, edible products for serving and eating a variety of dishes are already available in Europe, Asia, and America. We can read about them in popular science article and websites [287,288,289,290] or on the manufacturers’ websites. As mentioned earlier, tableware may be produced with wheat bran, and now bran from other cereals is of interest as a potential raw material [276]. The large sector of edible tableware and cutlery refers to baked items. The producers mostly declare that their edible soup bowls and cups as well as cutlery contain no chemicals, preservatives, fat, emulsifiers, artificial coloring, or milk products. Bakey’s cutlery is made from rice, wheat, and millet-based spoons [288,291]. Company Edibles by Jack [292] offers 18 different flavors (coconut curry, wasabi sesame, gingerbread, cranberry, and cornbread, just to mention some) of spoons in normal and mini-size. Another company, Edible Pro [293], is providing not only spoons but also baked biscuit cups and bowls. Recently, Dordevic and colleagues [294] presented nutrition values and mechanical properties of baked spoons made of grape, proso millet, wheat, xanthan, palm oil, and water in different proportions. They have concluded that despite the recapture for the cake, the baking temperature also impacted the textural properties of prepared items. To sum this up, the general trends observed for baked products, are the use of more sustainable raw materials, in meaning of cereals cultivated with lower water demand (using millet instead of rice or wheat) and baking as an alternative of more energy-consuming injection or extrusion processes. Natural colorants from vegetables (spinach, carrot, beet root) and spices such as turmeric are more commonly used.

Another sub-category of edibles is seaweed-originated products. Edible colored cups made of seaweed with a 30-day estimated time of biodegradation, known as Ello Jello cups, have been produced by Evoware [295]. Seaweed-based straws are produced by Loliware [296]. Recently, a very interesting product in form of sachets in the range of 15–100 mL, named Ooho, was presented. It is a waterproof film made from seaweed to encase liquid. It was proven to work as a capsule for drinks (non-alcoholic beverages as well as alcohol) and ketchup. Notpla declares that if it is not eaten together with the drink, the film will biodegrade in 4–6 weeks without a trace [297,298].

The sweet concept of dessert spoons made with cane sugar has been developed by Candy Cutlery. They are available plain and also in coffee, vanilla, strawberry, and peppermint flavors. The company presented shot glasses made from sugar, which have gained popularity across Canada [299].

The use of tableware and cutlery that can be eaten with a meal seems to be right. However, three points deserve special attention in obtaining and maintaining such products:

- physical and mechanical parameters of these products such as the strength on flexural and resistant to leakage or changeable temperature,
- biological and chemical safety of ingredients included in their composition,
- the way they are produced, packaged, and transported to the customer.

The first of them is a necessary and obvious condition to be met because poorly selected physical and mechanical parameters will contribute to the creation of defective goods that are unacceptable by consumers or will not allow for the production of items in 3D. Such conditions are described by Buxoo et al. [274] and Olt et al. [276], who designed the cup and plate using bran, peels, and leaves.

On the other hand, the microbiological and chemical safety of these products are not so obvious anymore. Natural biopolymers are generally non-toxic, but in order to increase the rheological properties, various plasticizers are introduced. In order to increase the tightness of cups or plates, additional coatings are also used. These ingredients are not always neutral to human health; therefore, they should be considered in the case of tableware or cutlery intended for consumption. When edible products (e.g., cups, plate, spoons) are ingested, they must be microbiologically safe. Production from agro-food waste carries the risk of the introduction of various microorganisms. Often, they may not survive processing, especially high temperatures, which are used in production. However, due to the fact that they are products from natural biopolymers and are biodegradable, they can be easily inhabited by microorganisms. Therefore, such products should be tested like food, in accordance with applicable standards. Any bacterial (especially *Enterobacteriaceae, Staphylococcus aureus*), yeast, and mold contamination should be identified.

The last important bullet point refers to the safety of edible packaging and technical challenges that still need to be overcome before they become more widespread. Moisture, heat, and microorganisms are the main concerns, making the long-term storage and transport of edible tableware and cutlery a hurdle, and additional packaging has to be used routinely to preserve them.

**Figure 5:**
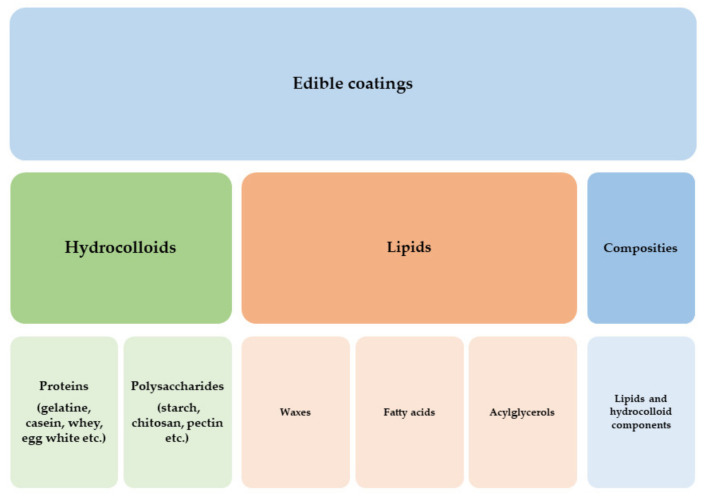
Edible coatings categorized due to their biopolymer components (based on [284,304]).

Currently, many scientific publications concern edible coatings, being a large category of food packaging materials [300,301,302]. Their growth took place already in 2002 (based on Web of Science); between 2015 and 2017, over 100 articles were written each year in this area; since 2018, over 200 have been published. The problem of edible coatings largely relates to edible packaging. These are thin films that form a coating on the product, which can also be used as food separating films. Most often, such products are made of natural polymers, mainly polysaccharides, as well as plant and animal proteins (Figure 5). The advantage of edible packaging is that it can be consumed together with the packaged product and is environmentally friendly [303]. In the food industry, edible casings are used in meat, fish, fruit, vegetable, and dairy processing.

## 6. Conclusions and Future Perspectives

Over the past several decades, plastic dishes and cutlery have been mass-produced, becoming a product of everyday use. An exceptional and still increasing number of plastic products, due to their low cost, durability, and flexibility, reached 348 million tons and is expected to double by 2040 [12]. Plastic pollution became a major environmental issue due to a short lifetime of single-use food packaging and serving items. Today, the world realized that this material may cause a serious problem for the environment pollution and human health if it is not biodegradable. The main concern is directed to marine environments. Amongst litter found on European beaches, 80–85% states for non-degradable plastic and 50% refers to single-use items [1]. To reduce these numbers, clear labelling of single-use products and information on their biodegradability as well as (bio)plastic content, proper disposal methods, and environmental risk are the solutions proposed in legislation and postulated by many organizations to overcome this problem. It should be supported with the replacement of non-biodegradable plastic with new-generation materials. Products made with multiple materials (such as multilayered items) should be modified or reconstructed in such a way that they will ensure simplified separation of the materials collected separately (for better recyclability).

What is more, the improvement of the garbage collection systems (with better separation of recyclable materials), providing higher recycling rates, should be implemented broadly. The diversity of materials and their varying susceptibility to biodegradation makes it difficult to make simple, general decisions on how to utilize them in a cost-effective and environmentally friendly way. The joint utilization of products made of multiple materials as well as co-utilization of conventional polymers and modern biomaterials is a challenge which should be faced in overcoming years. There is still a high risk that although the governments incorporate proper regulations and many initiatives are taken to educate consumers to make people aware about their personal responsibility in the pollution problem, without their commitment and goodwill, the problem of overconsumption and overproduction of disposable tableware and cutlery will not disappear. Summing up, the awaking of the social awareness on how to counteract the global pollution by personal shopping choices, and what seems to be more important, the new trends observed in the production of single-use products, developed due to the increasing responsibility of their producers, can decrease the impact by single-use products on the natural environment and human health. Increasing demand for more sustainable products boosts the research on new types of materials including disposable tableware and cutlery to design and develop more eco-friendly products used in everyday life.

## Data Availability

The data presented in this study are available on request from the corresponding author.
